# Single-Cell RNA Sequencing Reveals the Difference in Human Normal and Degenerative Nucleus Pulposus Tissue Profiles and Cellular Interactions

**DOI:** 10.3389/fcell.2022.910626

**Published:** 2022-07-07

**Authors:** Zhencong Li, Dongping Ye, Libing Dai, Yude Xu, Hao Wu, Wei Luo, Yiming Liu, Xiguan Yao, Peigeng Wang, Haixiong Miao, Jiake Xu, Weiguo Liang

**Affiliations:** ^1^ Guangzhou Red Cross Hospital, Guangzhou Red Cross Hospital of Jinan University, Guangzhou, China; ^2^ School of Biomedical Sciences, The University of Western Australia, Perth, WA, Australia

**Keywords:** nucleus pulposus cells, single-cell sequencing, intervertebral disc degeneration, cellular mapping, cellular interaction

## Abstract

**Background:** The nucleus pulposus is a constituent structure of the human intervertebral disc, and its degeneration can cause intervertebral disc degeneration (IDD). However, the cellular and molecular mechanisms involved remain elusive.

**Methods:** Through bioinformatics analysis, the single-cell transcriptome sequencing expression profiles of human normal nucleus pulposus (NNP) cells and human degenerative nucleus pulposus (DNP) cells were compared to clarify the transcriptome differential expression profiles of human NNP and DNP. The single-cell sequencing results of the two samples were analyzed using bioinformatics methods to compare the differences in histiocytosis between human NNP and DNP, map the histiocytes of NNP and DNP, perform cell differentiation trajectories for the cell populations of interest and predict cell function, and explore their heterogeneity by pathway analysis and Gene Ontology analysis.

**Results:** Nine cell types were identified, which were chondrocyte 1, chondrocyte 2, chondrocyte 3, chondrocyte 4, chondrocyte 5, endothelial, macrophage, neutrophil, and T cells. Analysis of the proportion of chondrocytes in different tissues revealed that chondrocyte 1 accounted for a higher proportion of NNP cells and highly expressed COL2A1 compared with DNP cells; chondrocyte 2, chondrocyte 3, chondrocyte 4, and chondrocyte 5 accounted for a higher proportion of DNP cells compared with NNP cells. Among them, chondrocyte 2 was an inhibitory calcified chondrocyte with high expression of MGP, chondrocytes 3 were fibrochondrocytes with high expression of COL1A1, chondrocytes 4 were chondrocytes that highly express pain inflammatory genes such as PTGES, and chondrocytes 5 were calcified chondrocytes with high expression of FN1 (chondrocytes 4 and chondrocytes 5 were found for the first time in a study of single-cell transcriptome sequencing of disc tissue). Cell trajectory analysis revealed that chondrocyte 1 was at the beginning of the trajectory and chondrocyte 3 was at the end of the trajectory, while chondrocyte 5 appeared first in the trajectory relative to chondrocyte 2 and chondrocyte 4.

**Conclusion:** After functional identification of the specifically expressed genes in five chondrocytes, it was found that chondrocyte 1 was a chondrocyte with high expression of COL2A1, COL9A2, COL11A2, and CHRDL2 in a high proportion of NNP cells, and chondrocyte 3 was a fibrochondrocyte with high expression of COL1A1, COL6A3, COL1A2, COL3A1, AQP1, and COL15A1 in an increased proportion during nucleus pulposus cell degeneration. Through cell trajectory analysis, it was found that chondrocytes 5 specifically expressing FN1, SESN2, and GDF15 may be the key cells leading to degeneration of nucleus pulposus cells. Chondrocytes 2 expressing MGP, MT1G, and GPX3 may play a role in reversing calcification and degeneration, and chondrocytes 4 expressing PTGES, TREM1, and TIMP1 may play a role in disc degeneration pain and inflammation.

## 1 Introduction

Intervertebral disc degeneration (IDD), as a common middle and senile disease, is extremely common, with an estimated global prevalence of about 10%, causing miserable and economic losses due to pain, limited mobility, and absenteeism. Of the 291 diseases studied in the Global Burden of Disease 2010, it ranked highest in terms of overall disability (Croft et al., 1995; Hoy et al., 2014; Wynne-Jones et al., 2014). Degenerative lumbar disc disease (DDD) refers to a series of clinical symptoms such as lower back pain, unilateral lower limb or lower limb pain, and numbness (Izzo et al., 2015). The pain and numbness of unilateral lower limb or lower limb are caused by different degrees of degenerative changes in various parts of lumbar intervertebral disc (annulus fibrosus, nucleus pulposus, and cartilage endplate), especially nucleus pulposus, due to rupture of annulus fibrosus, herniation of nucleus pulposus tissue from the rupture site out of posterior or spinal canal, and compression of corresponding spinal nerve roots. Lumbar disc degeneration characterized by late symptoms of progressive structural failure and disc aging is strongly associated with an increased risk of low back pain (Luoma et al., 2000). Genetic factors such as polymorphisms in genes encoding type I, IX, and XI collagen and polyproteoglycan and lifestyle such as lack of exercise and night shift work are associated with the development of IDD (Elfering et al., 2002; Feng et al., 2016). Surgery remains the mainstay of treatment for IDD. However, repeated surgery after the initial surgery due to adjacent or same disc recurrence can lead to serious functional complications. The use of analgesic drugs can help relieve symptoms but does not prevent the occurrence of IDD. Either the transcriptomic characteristics of nucleus pulposus tissue at single-cell resolution or the identification of novel chondrocytes can promote the understanding of DDD. More in-depth study of this genetic information may open a new window for future therapeutic interventions against degenerative diseases of the lumbar intervertebral disc. Therefore, it is essential to gain insight into the cell population changes in the NNP during degeneration and the related biological mechanisms.

Given that conventional bulk RNA sequencing (RNA-seq) is based on the assumption that each cell equally expresses each gene, it is not possible to accurately describe the heterogeneity of human NNP tissue and DNP tissue cells at the cell type level. With advances in scRNA-seq technology, heterogeneous tissues can be delineated at the single-cell level (Potter, 2018). This technology allows for massively parallel characterization of thousands of cells at the transcriptome level and can better account for cell–cell interactions. However, relatively few research results have been published on the single-cell transcriptome of the contrast between human NNP and DNP. The exact cellular and molecular mechanisms behind disc degeneration remain elusive. In order to explore the biological processes related to the new cellular processes and degeneration in nucleus pulposus cells, we used scRNA-seq to investigate cellular heterogeneity among different nucleus pulposus tissues, performed a relatively comprehensive single-cell transcriptome profiling of nucleus pulposus tissue, and identified gene features and cellular dynamics associated with nucleus pulposus cell degeneration. Our findings broaden the understanding of the biological network of nucleus pulposus cells and provide a theoretical basis for future treatment of disc degeneration.

## 2 Materials and Methods

### 2.1 Patient and Sample Collection

This work was approved by Guangzhou Red Cross Hospital affiliated to Jinan University and complied with all relevant ethical regulations. Patients who used clinical and cellular data for this study provided informed consent. In this study, DNP samples were isolated from an 81-year-old patient diagnosed with lumbar disc herniation/lumbar disc degeneration and NNP samples were isolated from an 11-year-old patient diagnosed with acute spinal cord injury. Fresh specimens collected at the time of surgical resection were collected in MACS tissue storage solution (Miltenyi Biotec, Germany) and transported to the laboratory as soon as possible.

### 2.2 Preparation of the Single-Cell Suspension

Tissue was surgically minced on a laboratory sterile table, and tissue fragments were preserved in MACS tissue storage until processing.

The tissue samples were processed as described below. Briefly, samples were first washed with phosphate-buffered saline (PBS), minced into small pieces (approximately 1 mm^3^) on ice, and enzymatically digested with 500 U/ml collagenase I (SangonBiotech), 150 U/ml collagenase II (SangonBiotech), 50 U/ml collagenase IV (SangonBiotech), 0.1 mg/ml hyaluronidase (SangonBiotech), 30 U/ml DNaseI (SangonBiotech), and 5% Fetal Bovine Serum Origin South America (Yeasen) for 60 min at 37°C, with agitation. After digestion, samples were sieved through a 70 μm cell strainer, and centrifuged at 300 g for 5 min. After washing with PBS containing 0.04% BSA, the cell pellets were re-suspended in PBS containing 0.04% BSA and re-filtered through a 35 μm cell strainer. Dissociated single cells were then stained for viability assessment using Calcein-AM (Thermo Fisher Scientific) and Draq7 (BD Biosciences). The single-cell suspension was further enriched with a MACS dead cell removal kit (Miltenyi Biotec).

### 2.3 Preparation of Single-Cell Suspensions for Library Construction and scRNA Sequencing

BD Rhapsody system was used to capture the transcriptomic information of the nucleus pulposus tissue sample single cells. Single-cell capture was achieved by random distribution of a single-cell suspension across >200,000 microwells through a limited dilution approach. Beads with oligonucleotide barcodes were added to saturation so that a bead was paired with a cell in a microwell. The cells were lysed in the microwell to hybridize mRNA molecules to barcode captured oligos on the beads. Beads were collected into a single tube for reverse transcription and ExoI digestion. Upon cDNA synthesis, each cDNA molecule was tagged on the 5′ end (i.e., the 3′ end of an mRNA transcript) with a unique molecular identifier (UMI) and cell barcode indicating its cell of origin. Whole-transcriptome libraries were prepared using the BD Rhapsody single-cell whole-transcriptome amplification (WTA) workflow including random priming and extension (RPE), RPE amplification PCR, and WTA index PCR. The libraries were quantified using a High Sensitivity DNA chip (Agilent) on a Bioanalyzer 2200 and the Qubit High Sensitivity DNA assay (Thermo Fisher Scientific). Sequencing was performed by Illumina Sequencer (Illumina, San Diego, CA, United States) on a 150 bp paired-end run.

### 2.4 Processing of scRNA-Sequencing Data

scRNA-seq data analysis was performed by NovelBio Bio-Pharm Technology Co., Ltd. with NovelBrain Cloud Analysis Platform. We applied fastp (Chen et al., 2018) with default parameter filtering the adaptor sequence and removed the low-quality reads to achieve clean data. UMI tools (Smith et al., 2017) were applied for single-cell transcriptome analysis to identify the cell barcode whitelist. The UMI-based clean data were mapped to the human genome (Ensemble version 100) utilizing STAR mapping with customized parameters from UMI tools’ standard pipeline to obtain the UMIs counts of each sample. Cells contained over 200 expressed genes and mitochondria UMI rate below 20% passed the cell quality filtering, and mitochondria genes were removed in the expression table.

### 2.5 Cell Clustering Analysis, Visualization, and Annotation

Seurat package (version: 3.1.4, https://satijalab.org/seurat/) was used for cell normalization and regression based on the expression table according to the UMI counts of each sample and percent of mitochondria rate to obtain the scaled data. Since samples were processed and sequenced in batches, we used MNN (mutual nearest neighbor) to remove potential batch effect. Subsequently, the top 10 principles were used for tSNE construction and UMAP construction.

Utilizing graph-based cluster method (resolution = 0.8), we acquired the unsupervised cell cluster result based on the top 10 principles and we calculated the marker genes by the FindAllMarkers function with Wilcox rank-sum test algorithm under the following criteria: 1) lnFC > 0.25; 2) *p*-value < 0.05; 3) min. pct > 0.1. In order to identify the cell type detailed, the clusters of the same cell type were selected for re-tSNE analysis, graph-based clustering, and marker analysis.

### 2.6 Pseudo-Time Analysis

We applied the single-cell trajectories analysis utilizing monocle 2 (http://cole-trapnell-lab.github.io/monocle-release) using DDR-Tree and default parameter. Before monocle analysis, we selected marker genes of the Seurat clustering result and raw expression counts of the cell passed filtering. Based on the pseudo-time analysis, branch expression analysis modeling (BEAM Analysis) was applied for branch fate determined by gene analysis.

### 2.7 Cell Communication Analysis

To enable a systematic analysis of cell–cell communication molecules, we applied cell communication analysis based on the CellPhoneDB (Vento-Tormo et al., 2018), a public repository of ligands, receptors, and their interactions. Membrane, secreted, and peripheral proteins of the cluster of different time points were annotated. Significant mean and cell communication significance (*p*-value < 0.05) were calculated based on the interaction and the normalized cell-matrix achieved by Seurat normalization.

### 2.8 Single-Cell Regulatory Network Inference and Clustering (SCENIC) Analysis

To assess transcription factor regulation strength, we applied the single-cell regulatory network inference and clustering (pySCENIC, v0.9.5) (Aibar et al., 2017) and workflow, using the 20,000 motifs database for RcisTarget and GRNboost.

#### 2.8.1 QuSAGE Analysis (Gene Enrichment Analysis)

To characterize the relative activation of a given gene set such as pathway activation, we performed QuSAGE (2.16.1) (Yaari et al., 2013) analysis.

#### 2.8.2 Coregulated Gene Analysis

To discover the gene coregulation network, the find_gene_modules function of monocle 3 (Cao et al., 2019) was used with the default parameters.

#### 2.8.3 CytoTRACE Analysis

We applied CytoTRACE analysis for predicting differentiation state with default parameter (Gulati et al., 2020).

### 2.9 Differential Gene Identification Between Normal and Degenerative Nucleus Pulposus Cells (RT-qPCR)

TNFα can cause inflammatory and degeneration of nucleus pulposus cells (Gabr et al., 2011). After NNP cells were stimulated with TNFα at a concentration of 25 ng/ml for 0, 24, 48, 72, and 96 h, the expression levels of COL2A1, MGP, COL1A1, PTGES, TIMP1, and FN1 genes in the cells were detected by RT-qPCR. The differences in high expression hallmark genes between human NNP and DNP cell populations were verified according to the changes in target gene expression levels after TNFα treatment.

### 2.10 Statistical Analysis

All statistical analysis and figures were generated using R software (version 3.6.3). A *p*-value < 0.05 was considered statistically significant.

## 3 Results

### 3.1 Cellular Contribution

**FIGURE 1:**
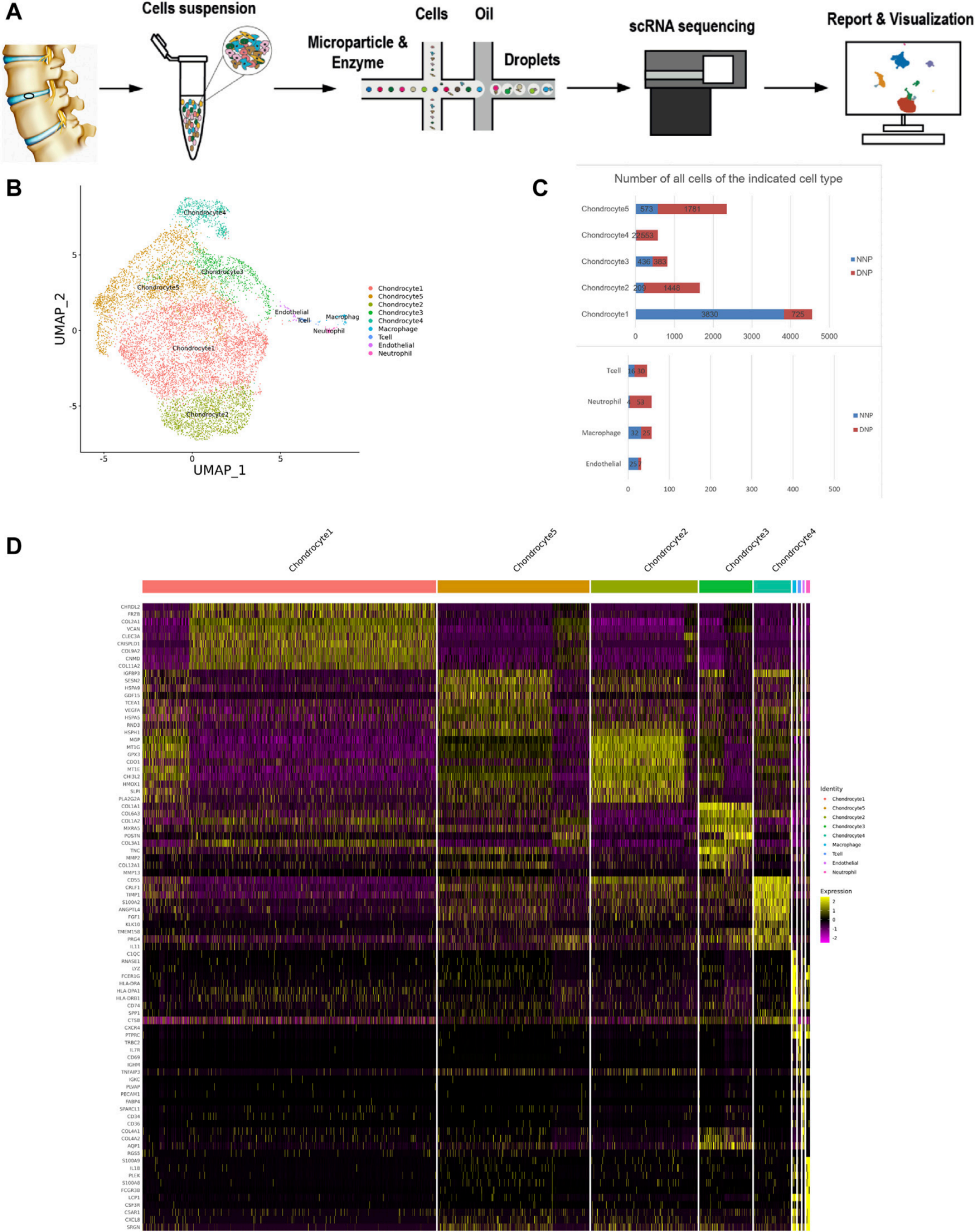
ScRNA-seq cluster analysis of two nucleus pulposus (NNP + DNP) cells. **(A)** The workflow for collection and processing of nucleus pulposus specimens for scRNA-seq. **(B)** UMAP plots of 10152 cells, showing the nine major cell types in nucleus pulposus. **(C)** Total number of cells with stacked column chart for specified cell type. **(D)** Heatmap showing and highlighting differentially expressed genes for each cluster.

**FIGURE 2:**
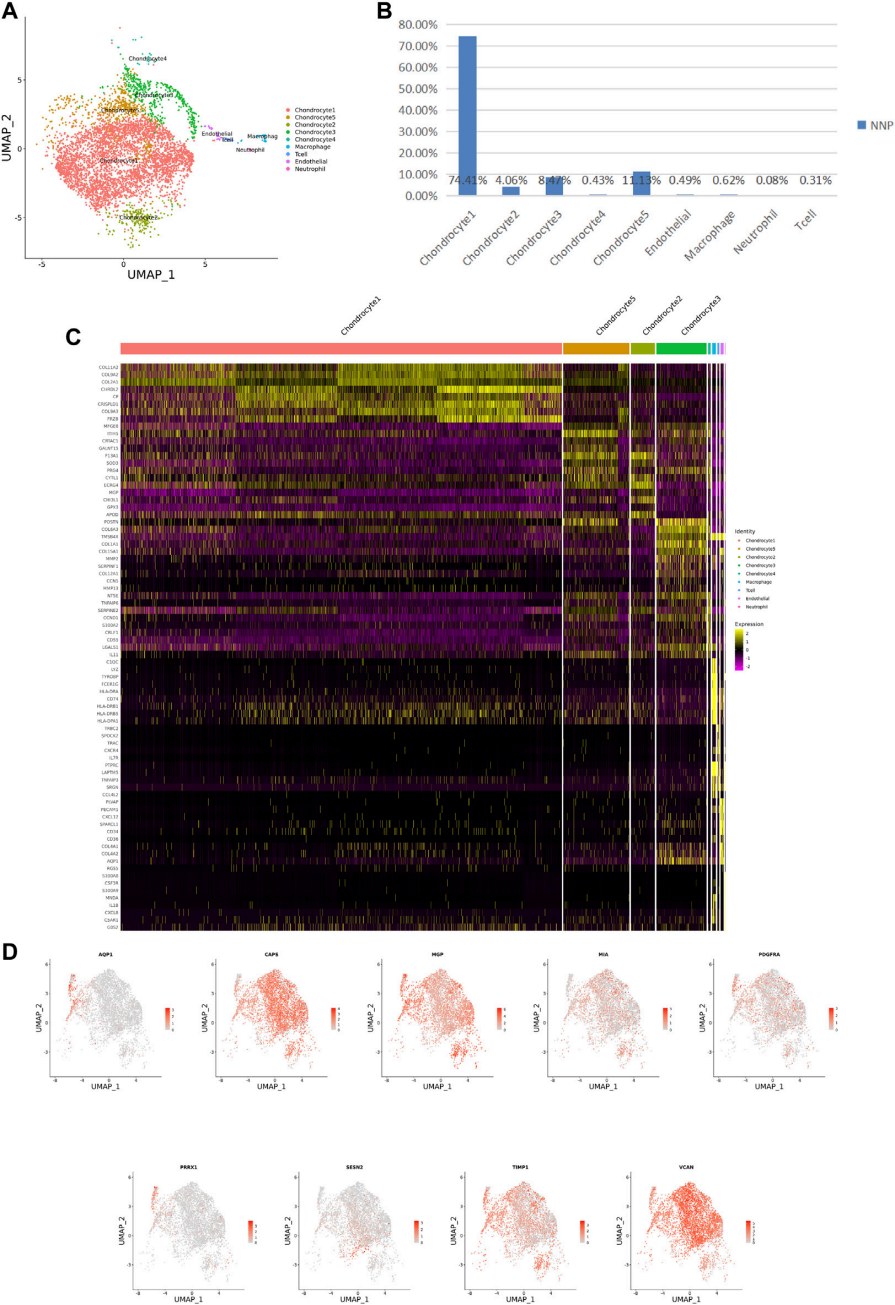
ScRNA-seq cluster analysis of NNP cells. **(A)** UMAP plots of 5,147 cells, showing the nine major cell types in NNP. **(B)** Total number of NNP cells with stacked column chart for specified cell type. **(C)** Heatmap showing and highlighting differentially expressed genes for each cluster. **(D)** UMAP plots showing the expression of representative well-known markers for each cell type in chondrocyte (NNP).

**FIGURE 3:**
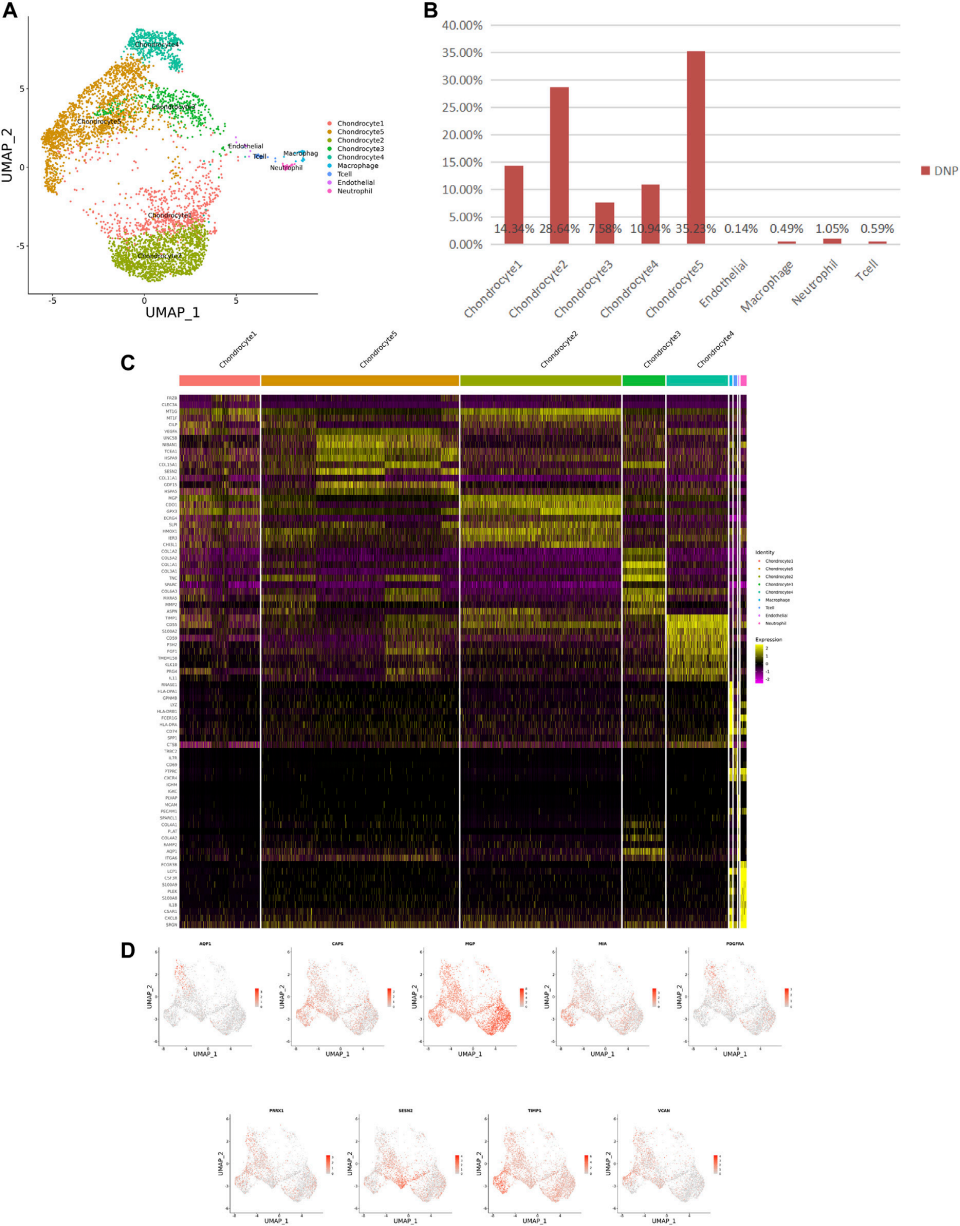
ScRNA-seq cluster analysis of DNP cells. **(A)** UMAP plots of 5,005 cells, showing the nine major cell types in DNP. **(B)** Total number of DNP cells with stacked column chart for specified cell type. **(C)** Heatmap showing and highlighting differentially expressed genes for each cluster. **(D)** UMAP plots showing the expression of representative well-known markers for each cell type in chondrocyte (DNP).

After the single-cell sequencing experimental process (Figure 1A), the experimental data were obtained. In this study, single-cell transcriptome sequencing was performed on two samples and the total cell number was obtained to be 10152 (Figure 1C). Among them, the total number of effective cells captured by human NNP tissue samples was 5,147 (Figure 2B); the total number of effective cells captured by human degenerative nucleus pulposus tissue samples was 5,005 (Figure 3B). After the initial quality control assessment, single-cell transcriptomics data from 10,152 valid cells from both samples were used for further analysis. The results of the distribution after dimension reduction showed that cells with closer distances indicated more similar gene expressions. Nine major clusters of cells were identified (Figure 1B,D, 2C, 3C): chondrocyte 1, chondrocyte 2, chondrocyte 3, chondrocyte 4, chondrocyte 5, endothelial, macrophage, neutrophil, and T cell. Chondrocyte 2 found in this study has not been previously studied with clear definitions, and newly discovered chondrocyte 4 and chondrocyte 5 have not been reported.

### 3.2 Cellular Heterogeneity Between Human Normal Nucleus Pulposus Tissue and Human Degenerative Nucleus Pulposus Tissue

After identifying the markers of the above five chondrocyte populations obtained by reduced-dimensional cluster analysis, comparative analysis of human NNP tissue cells populations and DNP cells populations revealed that chondrocytes 1 (74.41%) accounted for a higher proportion of NNP tissue compared with DNP tissue; chondrocytes 2 (28.93%), chondrocytes 4 (11.05%), and chondrocytes 5 (35.58%) accounted for a higher proportion of DNP tissue compared with NNP tissue (Figures 2A,B, Figures 3A,B). In human DNP tissue, the ratio of chondrocyte 3 and chondrocyte 1, was much higher than that in human NNP tissue.

**FIGURE 4:**
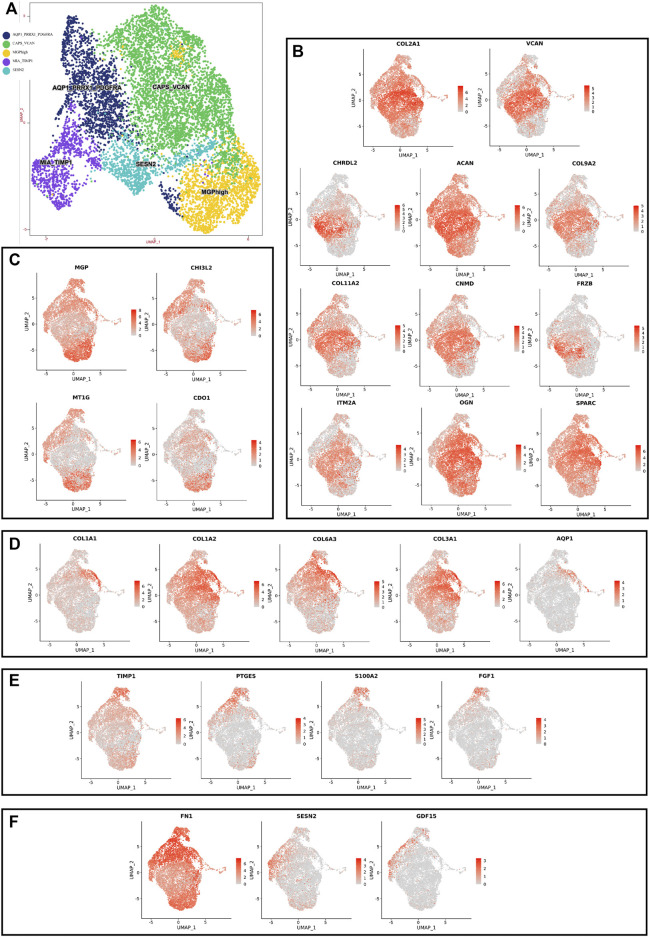
scRNA-seq clustering marker gene analysis of nucleus pulposus cells. **(A)** UMAP plots show the expression of representative well-known markers across cell types in chondrocyte l of nucleus pulposus cells. **(B)** UMAP plot of chondrocyte 1 subtype, the expression level (up), and the AUC of the estimated regulon activity of these transcription factors (down). **(C)** UMAP plot of chondrocyte 2 subtype, the expression level (up), and the AUC of the estimated regulon activity of these transcription factors (down). **(D)** UMAP plot of chondrocyte 3 subtype, the expression level (up), and the AUC of the estimated regulon activity of these transcription factors (down). **(E)** UMAP plot of chondrocyte 4 subtype, the expression level (up), and the AUC of the estimated regulon activity of these transcription factors (down). **(F)** UMAP plot of chondrocyte 5 subtype, the expression level (up), and the AUC of the estimated regulon activity of these transcription factors (down).

Although human NNP tissue and human DNP tissue are composed of the same cell type, the proportion of highly expressed genes in the whole nucleus pulposus tissue is also heterogeneous due to the different proportion of each cell ethnic group in the two tissues (Figures 2D, 3D, Figure 4).

Using unbiased clustering of the data, combined with the investigated literature and databases, based on known cell markers, the following chondrocyte types were determined: 1) chondrocyte 1 and the mainly expressed characteristic genes were COL2A1, COL9A2, COL11A2, and CHRDL2; and 2) chondrocytes 3: fibrochondrocytes and the mainly expressed characteristic genes were COL1A1, COL6A3, COL1A2, COL3A1, and AQP1 (Gunja and Athanasiou, 2007; Baryawno et al., 2019). Moreover, the other three cell types have not been clearly defined in previous studies; 3) chondrocytes 2: calcification inhibits chondrocytes, and the mainly expressed characteristic genes were MGP, MT1G, GPX3, CDO1, MT2A, and CLU (Schurgers et al., 2007); 4) chondrocytes 4: inflamed chondrocytes, and the mainly expressed characteristic genes were PTGES, TREM1, TIMP1, and TIMP3; and 5) chondrocytes 5: calcifying chondrocyte, and the mainly expressed characteristic genes were FN1, SESN2, GDF15, and COL15A1.

### 3.3 Single-Cell Trajectory Branch Points Correspond to Chondrocyte Differentiation

**FIGURE 5:**
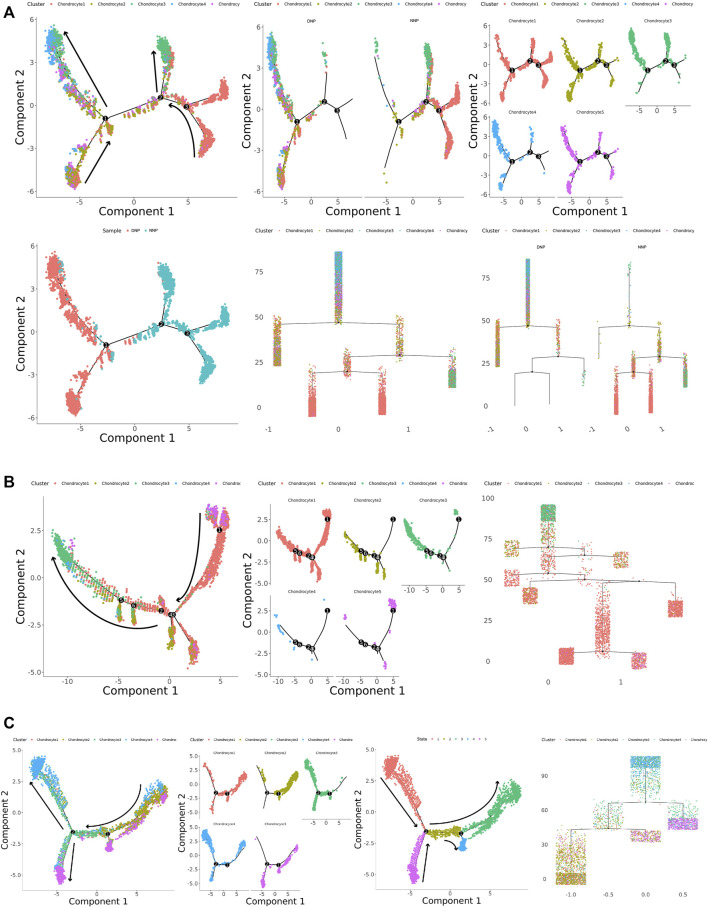
Monocle pseudo-time trajectory. **(A)** Monocle pseudo-time trajectory showing the progression of chondrocyte 1, chondrocyte 2, chondrocyte 3, chondrocyte 4, and chondrocyte 5 in nucleus pulposus cells. **(B)** Monocle pseudo-time trajectory showing the progression of chondrocyte 1, chondrocyte 2, chondrocyte 3, chondrocyte 4, and chondrocyte 5 in NNP cells. **(C)** Monocle pseudo-time trajectory showing the progression of chondrocyte 1, chondrocyte 2, chondrocyte 3, chondrocyte 4, and chondrocyte 5 in DNP cells.

To investigate the differentiation process and corresponding gene expression of nucleus pulposus chondrocytes, we constructed a monocle pseudo-time trajectory of chondrocytes from five cell populations, containing the trajectory of all cells in human NNP tissue and DNP tissue (Figure 5A), the trajectory of human NNP tissue (Figure 5B), and the trajectory of human DNP tissue (Figure 5C).

The trace showed that chondrocyte 1 accumulated at the tip of the trace, while chondrocyte 3 accumulated at the tail end of the trace (Figure 5A). FN1 + Chondrocyte 5 appears at the tip of the trajectory concomitant with chondrocyte 1, and MGP + Chondrocyte 2 participates in the trajectory as a branch. In human NNP tissue, PTGES + Chondrocyte 4 was less numerous and hardly involved in the trajectory (Figure 5B). In human DNP tissue, the number of PTGES + Chondrocyte 4 increased substantially and was accompanied by fibrochondrocytes chondrocyte 3 at the posterior end of the trajectory. MGP + Chondrocyte 2 and FN1 + Chondrocyte 5 appeared at the tip of the trajectory at the same time, accompanied by chondrocyte 1. When the number of chondrocyte 1 decreased, the number of fibrochondrocytes chondrocyte 3 increased (Figure 5C). Cell relationships were analyzed by cellular gene expression, and by establishing a new trajectory that analyzed the cells independently (Figure 5C), it was found that chondrocyte 1, fibrochondrocytes chondrocyte 3, and PTGES + Chondrocyte 4 formed an independent tail end, head end, and tail end of the trajectory, respectively. While FN1 + Chondrocyte5 formed a cell fate key node in the trajectory with chondrocyte 1 when it participated in the trajectory in the form of branches, MGP + Chondrocyte 2 appeared successively, and when it experienced the next cell fate node, fibrochondrocytes chondrocyte 3 and PTGES + Chondrocyte 4 appeared and formed the tail ends, respectively.

In summary, chondrocyte 1 is the normal cell subtype of NNP tissue, and chondrocyte 3 is the fibrochondrocyte subtype of DNP tissue. Chondrocyte 5 may be a key cell leading to degeneration of nucleus pulposus cells. While chondrocyte 2 plays a role in reversing calcification and degeneration, chondrocyte 4 plays a role in disc degeneration pain and inflammation-related effects. During the development of degeneration of human nucleus pulposus tissue, a large number of normal chondrocytes 1 are affected by chondrocytes 2, chondrocytes 4, and chondrocytes 5 when differentiating into fibrochondrocytes 3, producing changes in nucleus pulposus tissue and the occurrence of pain, accumulation of numerous chondrocytes 3, or the end result of degeneration of human nucleus pulposus tissue occurs.

### 3.4 Differential Gene Identification Between Normal and Degenerative Nucleus Pulposus Cells (RT-qPCR)

**FIGURE 6:**
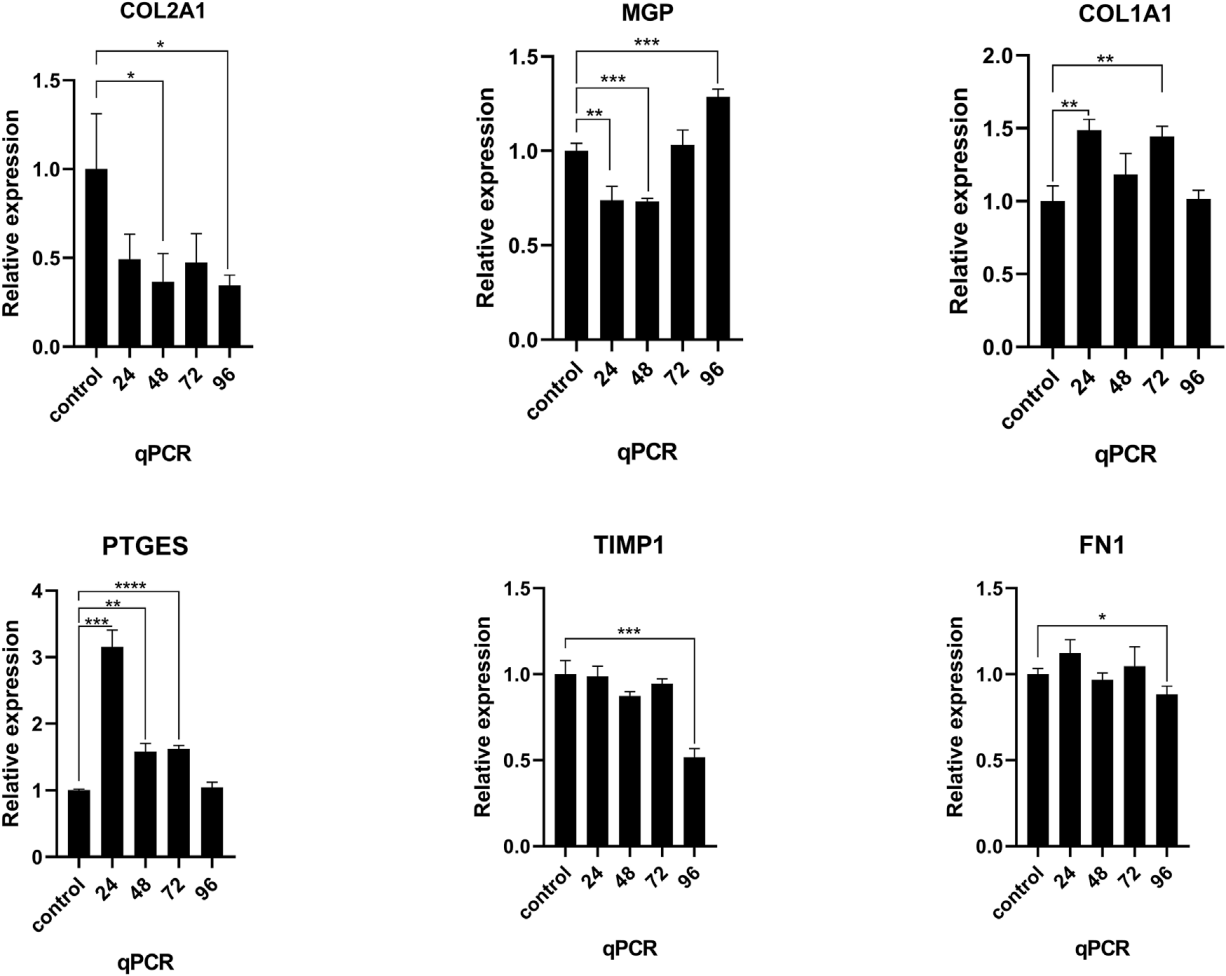
RT-qPCR was used to detect the mRNA expression of COL2A1, MGP, COL1A1, PTGES, TIMP1, and FN1 in human NNP cells treated with TNFα for different time periods. *p < 0.05, *p < 0.01, *p < 0.001, *p < 0.0001.

After NNP cells were stimulated with TNFα at a concentration of 25 ng/ml for different times 0, 24, 48, 72, and 96 h, RNA expression was measured using RT-qPCR (Figure 6). Compared with NNP cells at 0 h in the control group, the expression level of COL2A1 decreased after drug treatment and decreased to the lowest level at 96 h. There was a significant difference in the expression level between 48, 96, and 0 h (*p* < 0.05). The expression level of COL1A1 was increased, and the expression level was the highest at 24 h. There was a significant difference in the expression level between 24, 72, and 0 h (*p* < 0.01). PTGES expression was increased and was the highest at 24 h. There was a significant difference in expression between 24, 48, 72, and 0 h (*p* < 0.0001). MGP expression increased after decreasing, or was activated due to the conditions required for the gene to reverse calcification function, with a significant difference in expression between 24, 48, and 96, and 0 h (*p* < 0.001). However, the expression levels of TIMP1 and FN1 genes did not reach the expected results of the experiment (*p < 0.05,*p < 0.01,*p < 0.001,*p < 0.0001).

### 3.5 Complex Intercellular and Molecular Interaction Networks in Nucleus Pulposus Cells

**FIGURE 7:**
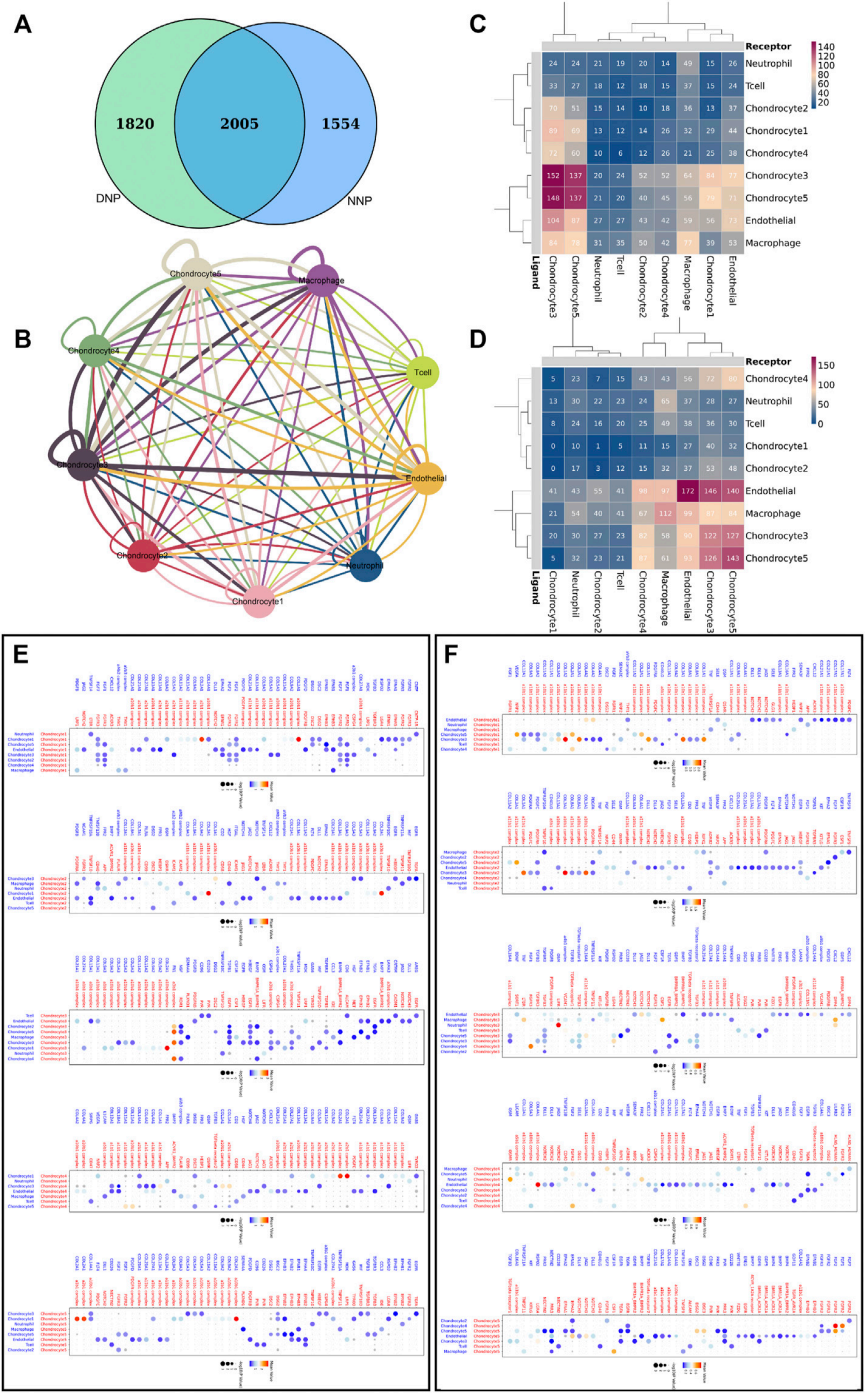
Cell–cell communication network of CellPhoneDB in the nucleus pulposus cells. **(A)** Venn diagram of genes related to cell communication, and NNP is related to DNP cell cellphone relations number in our data. **(B)** Interaction network constructed by CellPhoneDB. Thicker lines indicate more interaction with other types of cells. **(C)** Heatmap showing the number of potential ligand–receptor pairs among the predicted cell types in NNP. **(D)** Heatmap showing the number of potential ligand–receptor pairs among the predicted cell types in DNP. **(E)** NNP cell ligand–receptor pairs detected with CellPhoneDB are shown in a bubble plot. **(F)** DNP cell ligand–receptor pairs detected with CellPhoneDB are shown in a bubble plot.

Genes related to cell communication with a number of 2005 were found expressed by nucleus pulposus cells (Figure 7A). CellPhoneDB analysis was used to calculate the number of ligand–receptor pairs in all cell types of human NNP tissue and DNP tissue in our data (Figures 7C,D). These ligand–receptor relationships are shown in Figure 7B. Five chondrocyte populations in each of the two nucleus pulposus tissues were selected as receptor target cells, and their ligand–receptor relationships were identified (Figures 7E,F). All of these ligand–receptor pairs are expressed individually. All chondrocytes can bind to the ligand COL2A1 as a receptor, which has the function of calcium metal binding. Type II collagen is specific for cartilage tissue. It is essential for normal embryonic development, linear growth, and cartilage resistance to stress in the skeleton, while its receptors in various types of chondrocyte populations are as follows: chondrocyte 1 (a10b1 complex, a2b1 complex), chondrocyte 2 (a11b1 complex), chondrocyte 3 (a10b1 complex), chondrocyte 4 (a2b1 complex, a1b1 complex), and chondrocyte 5 (a10b1 complex, a2b1 complex, and a1b1 complex). In human NNP tissue, which has the largest proportion of chondrocytes 1, the a10b1 complex and the a2b1 complex are largely activated. In contrast, in human degenerative nucleus pulposus tissue, the number of activations of a10b1 complex, a2b1 complex, a1b1 complex, and a11b1 complex has a large advantage. The a3b1 complex receptor of chondrocyte 3 can bind to FN1 ligands of multiple cells such as chondrocyte 5, chondrocyte 3, chondrocyte 4, and chondrocyte 2. Chondrocyte 3 may have a relationship with fibrocalcifications. Combined with gene function analysis, chondrocyte 3, which has an increasing proportion to the number of chondrocyte 1, may have a direct relationship with fibrocalcification during degeneration of human nucleus pulposus tissue.

**FIGURE 8:**
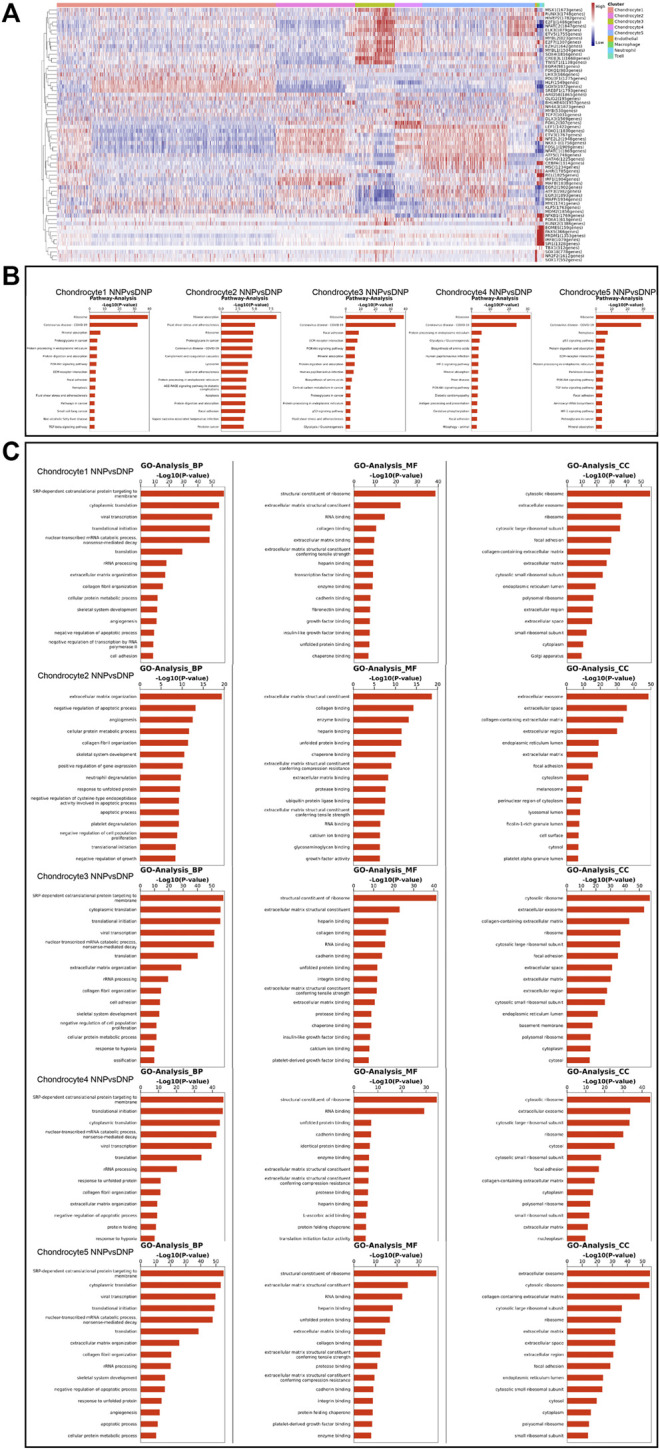
Gene Regulatory Network, Gene Ontology analysis, and pathway analysis in the nucleus pulposus cells. **(A)** Heatmap of the AUC scores of transcription factor expression regulation by SCENIC. **(B)** A barplot showing the signaling pathways of interest in NP cell as detected by CellChat. **(C)** A barplot showing the Gene Ontology analysis of NP cell including biological process, molecular function, and cellular component.

Single-Cell Regulatory Network Inference and Clustering (SCENIC) analysis revealed transcriptional regulatory network-related genes in different cell populations (Figure 8A): chondrocyte 1: SOX9 (1972gene), SREBF1 (1793genes), and HLF (1549gens); chondrocyte 2: NFE2L2 (1948genes), FOSL1 (1909genes), IRF1(1904genes), NFATC1 (1869genes), MAFB (1838genes), FOXO1 (1830genes), ETV3 (1767genes), and NKX3-1 (1758genes); chondrocyte 3: ELK3 (1879genes), NFATC2 (1847genes), SOX4 (1816genes), CREB3L1 (1668genes), MYBL1 (1504genes), E2F1 (1486genes), E2F7 (1307genes), and TWIST1 (1138genes); chondrocyte 4: LEF1 (1422genes) and FOXL2 (307genes); and chondrocyte 5: FOSL1 (1909genes), LEF1 (1422genes), CEBPA (1314genes), MSC (1234genes), and GATA6 (1225genes). The results revealed that the transcriptional status of nucleus pulposus chondrocytes in different states has different upstream transcription factors and cofactors as well as downstream target genes composed of gene regulatory network (GRN). In human NNP tissue, which has the largest proportion of chondrocytes 1, the SOX9, SREBF1, and HLF are largely activated. In contrast, in human DNP tissue, the number of activations of NFE2L2, FOSL1, IRF1, ELK3, NFATC2, SOX4, LEF1, and FOSL1 has a large advantage.

Pathway analysis revealed the expression of signaling pathways with different degrees of enrichment in different cell populations (Figure 8B): chondrocyte 1: ribosome, mineral absorption, proteoglycans in cancer, focal adhesion, ferroptosis, fluid shear stress, and atherosclerosis; chondrocyte 2: mineral absorption, fluid shear stress and atherosclerosis, ribosome, proteoglycans in cancer, complement and coagulation cascades, lipid and atherosclerosis, apoptosis, and focal adhesion; chondrocyte 3: ribosome, focal adhesion, ECM–receptor interaction, PI3K-Akt signaling pathway, mineral absorption, p53 signaling pathway, fluid shear stress, and atherosclerosis; chondrocyte 4: ribosome, HIF-1 signaling pathway, mineral absorption, and PI3K-Akt signaling pathway; and chondrocyte 5: ribosome, ferroptosis, p53 signaling pathway, ECM–receptor interaction, PI3K-Akt signaling pathway, focal adhesion, and HIF-1 signaling pathway. This result revealed that each chondrocyte population of the nucleus pulposus has different degrees of enrichment of signal transduction pathways in different states.

In human NNP tissue, which has the largest proportion of chondrocytes 1, cellular pathways involved in mineral absorption, focal adhesion, and ferroptosis are largely activated. In contrast, the degree of activation of fluid shear stress, atherosclerosis-related pathway, complement, and coagulation cascades, apoptosis, focal adhesion, PI3K-Akt signaling pathway, mineral absorption, HIF-1 signaling pathway, ferroptosis, and p53 signaling pathway are more evident in human DNP tissue. Among them, mineral absorption, ferroptosis, fluid shear stress, atherosclerosis-related pathway, apoptosis, and focal adhesion may be important signaling pathways involved in the process of human nucleus pulposus tissue degeneration.

Gene Ontology analysis (GO analysis) assigned significant differential genes in nucleus pulposus chondrocytes to different functional classifications (Figure 8C). GO analysis can describe the function of genes from all aspects, while GO can be mainly divided into three main groups, biological process (BP), molecular function (MF), and cellular component (CC). GO analysis revealed different functions of nucleus pulposus chondrocytes in different ethnic groups. BP is closer to the biological phenotype of cell population. Through the analysis of highly expressed genes, it is found that the cell population with high proportion in human NNP tissue has the following cellular functions: collagen fibril organization, negative regulation of apoptotic process, and cell adhesion. However, the cell population, which accounts for a high proportion of human DNP tissue, has the following specific cellular functions including extracellular matrix organization, skeletal system development, translational initiation, and ossification.

## 4 Discussion

Degenerative disc disease is the leading cause of chronic back pain due to the aging population in the world (Vos et al., 2012). Various factors have been previously studied to determine the cause of degenerative disc disease and its pain generation pathway in an attempt to find strategies to reverse and prevent further degeneration or provide optimal pain management to reduce pain and suffering in patients with degenerative disc disease. Various genetic, environmental, biomechanical, and anatomical variations have been found to be associated with degenerative disc disease (Zawilla et al., 2014; Zhu et al., 2016; Wong et al., 2017). Past studies on the pathogenesis of degenerative disc disease mostly lie in the exploration of the overall pathological change direction of the tissue (Amelot and Mazel, 2018) with dysregulation of gene expressions and signaling pathways (Liang et al., 2014; Ye et al., 2015), and some possible disease evolution processes and mechanisms will be ignored, such as the role of the heterogeneity of single cells in the tissue during the pathogenesis in the disease. In the past, the pain caused by degenerative disc disease was considered to be discogenic, but no clear conclusion was made on the process of cell evolution and the change in cell population type when the degeneration of NNP tissue occurred.

In this study, we constructed a cell population map of human NNP tissue and human DNP tissues at the RNA level by integrating and clustering cells. Through the identification of classical markers, we dissected different cell types and their differentiation status and function and annotated chondrocyte associated with the disease. Compared with previous studies of pathological tissue specimens, this study further classifies cells at the genomic and transcriptomic levels and provides relevant clues at the cytological level for the pathogenesis of the disease through the dissection of cell differentiation trajectories.

Through cluster analysis of the results of this single-cell sequencing, this study found that the cells of human nucleus pulposus tissue are mainly divided into two categories: chondrocyte group and non-chondrocyte group. The chondrocyte population was the most abundant, including chondrocytes 1, chondrocytes 2 (inhibits calcified chondrocytes), chondrocytes 3 (fibrochondrocytes), chondrocytes 4 (painful inflammatory chondrocytes), and chondrocytes 5 (calcifying chondrocyte). Among them, chondrocytes 4 and chondrocytes 5 were the first to be discovered in a study related to single-cell sequencing of nucleus pulposus cells. Non-chondrocyte populations include endothelial, macrophage, neutrophil, and T cells. After the classification was defined by cell cluster analysis, it was found that the number and proportion of each cell population in human NNP tissues were different from those in human DNP tissues. Functional analysis of each cell population by Gene Ontology revealed that chondrocyte 1 may have cellular functions such as cartilage development, tissue homeostasis, and cartilage development involved in endochondral bone morphogenesis; chondrocyte 2 may have the cellular functions of cartilage development involved in endochondral bone morphogenesis, cartilage development involved in endochondral bone morphogenesis, negative regulation of bone mineralization, and metal-ion binding; chondrocytes 3 may have the cellular function of ossification, endochondral ossification, and skeletal system development; chondrocyte 4 may have the cellular function of prostaglandin biosynthetic process, regulation of acute inflammatory response, and inhibition of extracellular matrix degradation; and chondrocytes 5 may have cellular functions involved in fibrin clot formation.

With the NNP tissue, chondrocyte 1 accounted for a higher proportion of NNP cells, and with the DNP tissue, chondrocyte 2, chondrocyte 3, chondrocyte 4, and chondrocyte 5 accounted for a higher proportion of DNP cells. During the disease process, the population of human DNP cells with increased proportion may be the key to the outcome and development of degenerative disc disease.

To test the conjecture, cell trajectory analysis was performed on NNP and DNP tissue samples, respectively, and single-cell pseudo-timing analysis revealed that chondrocyte 1 was at the tip of the cell differentiation trajectory, chondrocyte 3 was at the end of the trajectory, while chondrocyte 5 appeared first in the trajectory relative to chondrocyte 2 and chondrocyte 4. Combined with the cell population-specific expression gene set, it is reasonable to believe that chondrocyte 1 is the normal cell subtype of NNP tissue, and chondrocyte 3 is the fibrochondrocyte subtype of DNP tissue. Chondrocyte 5 may be a key cell leading to degeneration of nucleus pulposus cells, while chondrocyte 2 may reverse calcification and degeneration and chondrocyte 4 may play a pain- and inflammation-related role in disc degeneration.

In order to verify the changes in the proportion of human NNP cells in the process of degeneration, human NNP cells treated with TNFα for different times were detected by RT-qPCR, and the expression levels of highly expressed specific genes, COL2A1, MGP, COL1A1, PTGES, TIMP1, and FN1 genes, in different cell populations were detected. After the addition of TNFα, COL2A1, COL1A1 and PTGES expression increased, and the differential changes in the expression of these genes were statistically significant. MGP expression first decreased and then increased, or because the condition required for the gene to reverse calcification function was activated. However, the expression levels of TIMP1 and FN1 genes did not reach the expected results of the experiment.

Studies have also found heterogeneity in signaling pathways between human NNP cells and DNP cells, as well as their own transcriptional regulatory network-related genes in different cell populations. Pathway analysis showed that different cell populations had different enrichment degrees of signaling pathway expression in human NNP and DNP cell populations. Gene Ontology analysis is also heterogeneous.

In this study, single-cell transcriptome sequencing and analysis were performed in NNP and DNP tissues, and the cell heterogeneity and cell differentiation trajectory in human NNP and DNP tissues were revealed for the first time at the transcriptome level. Among them, the composition and proportion of different cell populations provide important information for the study of the disease development process of nucleus pulposus tissue. We found that COL2A1 + Chondrocyte 1 is the main normal cell population of nucleus pulposus tissue; COL3A1 + Chondrocyte 3 is the main degenerative cell population of nucleus pulposus tissue; MGP + Chondrocyte 2 or chondrocytes can reverse calcification; PTGES + Chondrocyte 4 are related to inflammatory pain; and FN1 + Chondrocyte 5 or chondrocytes lead to calcification, and may also be the key cells leading to degeneration and fibrosis of nucleus pulposus tissue. The results of this research can provide an important reference for studying the pathogenesis of degenerative disc disease and deepen the understanding of chondrocyte diversity in nucleus pulposus tissue, providing a reference cell map for studying cartilage disease research.

## Data Availability

Supplemental material for this article can be found at https://www.ncbi.nlm.nih.gov/geo/query/acc.cgi?acc=GSE205535.
