# Corrigendum: Single-cell RNA sequencing reveals the difference in human normal and degenerative nucleus pulposus tissue profiles and cellular interactions

**DOI:** 10.3389/fcell.2022.1051707

**Published:** 2022-10-12

**Authors:** Zhencong Li, Dongping Ye, Libing Dai, Yude Xu, Hao Wu, Wei Luo, Yiming Liu, Xiguan Yao, Peigeng Wang, Haixiong Miao, Jiake Xu, Weiguo Liang

**Affiliations:** ^1^ Guangzhou Red Cross Hospital, Guangzhou Red Cross Hospital of Jinan University, Guangzhou, China; ^2^ School of Biomedical Sciences, The University of Western Australia, Perth, WA, Australia

**Keywords:** nucleus pulposus cells, single-cell sequencing, intervertebral disc degeneration, cellular mapping, cellular interaction

In the published article, there was an error in affiliation 1. Instead of “Guangzhou City Red Cross Hospital, The Fourth Affiliated Hospital of Medical College, Jinan University, Guangzhou, China”, it should be “Guangzhou Red Cross Hospital, Guangzhou Red Cross Hospital of Jinan University, Guangzhou, China”.

The authors apologize for this error and state that this does not change the scientific conclusions of the article in any way. The original article has been updated.

