# Corrigendum: Single-cell RNA sequencing reveals the difference in human normal and degenerative nucleus pulposus tissue profiles and cellular interactions

**DOI:** 10.3389/fcell.2023.1108743

**Published:** 2023-01-24

**Authors:** Zhencong Li, Dongping Ye, Libing Dai, Yude Xu, Hao Wu, Wei Luo, Yiming Liu, Xiguan Yao, Peigeng Wang, Haixiong Miao, Jiake Xu, Weiguo Liang

**Affiliations:** ^1^ Guangzhou Red Cross Hospital, Guangzhou Red Cross Hospital of Jinan University, Guangzhou, China; ^2^ School of Biomedical Sciences, The University of Western Australia, Perth, WA, Australia

**Keywords:** nucleus pulposus cells, single-cell sequencing, intervertebral disc degeneration, cellular mapping, cellular interaction

In the published article, there was an error in the **Data availability statement**. The correct Data Availability statement appears below.

“Supplemental material for this article can be found at https://www.ncbi.nlm.nih.gov/geo/query/acc.cgi?acc=GSE205535.”

The authors apologize for this error and state that this does not change the scientific conclusions of the article in any way. The original article has been updated.

